# Generation of populations of antigen-specific cytotoxic T cells using DCs transfected with DNA construct encoding HER2/neu tumor antigen epitopes

**DOI:** 10.1186/s12865-017-0219-7

**Published:** 2017-06-20

**Authors:** Maria Kuznetsova, Julia Lopatnikova, Julia Khantakova, Rinat Maksyutov, Amir Maksyutov, Sergey Sennikov

**Affiliations:** 1 Federal State Budgetary Scientific Institution “Research Institute of Fundamental and Clinical Immunology”, Yadrintsevskaya str., 14, Novosibirsk, 630099 Russia; 2 State Research Center of Virology and Biotechnology “VECTOR”, Koltsovo, Novosibirsk Region 630559 Russia

**Keywords:** Cytotoxic T cells, CTLs, Antigen-specific cells, Dendritic cells, HER2/neu, Tumor-associated antigen, Antitumor immune response

## Abstract

**Background:**

Recent fundamental and clinical studies have confirmed the effectiveness of utilizing the potential of the immune system to remove tumor cells disseminated in a patient’s body. Cytotoxic T lymphocytes (CTLs) are considered the main effectors in cell-mediated antitumor immunity. Approaches based on antigen presentation to CTLs by dendritic cells (DCs) are currently being intensively studied, because DCs are more efficient in tumor antigen presentation to T cells through their initiation of strong specific antitumor immune responses than other types of antigen-presenting cells. Today, it has become possible to isolate CTLs specific for certain antigenic determinants from heterogeneous populations of mononuclear cells. This enables direct and specific cell-mediated immune responses against cells carrying certain antigens. The aim of the present study was to develop an optimized protocol for generating CTL populations specific for epitopes of tumor-associated antigen HER2/neu, and to assess their cytotoxic effects against the HER2/neu-expressing MCF-7 tumor cell line.

**Methods:**

The developed protocol included sequential stages of obtaining mature DCs from PBMCs from HLA A*02-positive healthy donors, magnet-assisted transfection of mature DCs with the pMax plasmid encoding immunogenic peptides HER2 p369–377 (E75 peptide) and HER2 p689–697 (E88 peptide), coculture of antigen-activated DCs with autologous lymphocytes, magnetic-activated sorting of CTLs specific to HER2 epitopes, and stimulation of isolated CTLs with cytokines (IL-2, IL-7, and IL-15).

**Results:**

The resulting CTL populations were characterized by high contents of CD8^+^ cells (71.5% in cultures of E88-specific T cells and 90.2% in cultures of E75-specific T cells) and displayed strong cytotoxic effects against the MCF-7 cell line (percentages of damaged tumor cells in samples under investigation were 60.2 and 65.7% for E88- and E75-specific T cells, respectively; level of spontaneous death of target cells was 17.9%).

**Conclusions:**

The developed protocol improves the efficiency of obtaining HER2/neu-specific CTLs and can be further used to obtain cell-based vaccines for eradicating targeted tumor cells to prevent tumor recurrence after the major tumor burden has been eliminated and preventing metastasis in patients with HER2-overexpressing tumors.

**Electronic supplementary material:**

The online version of this article (doi:10.1186/s12865-017-0219-7) contains supplementary material, which is available to authorized users.

## Background

The minimal residual disease remaining after resection of the major tumor burden underlies the existing problems of tumor recurrence and metastasis, which increase the mortality and morbidity rates among cancer patients. In this connection, there is obviously a need for the development of new technologies that can improve the recognition and elimination of single cancer cells remaining in a patient’s body after radiation therapy, chemotherapy, or surgical resection.

Currently, cytotoxic T cells are considered the main effectors in cell-mediated antitumor immunity. The presence, number, and adequate function of antitumor cytotoxic T lymphocytes (CTLs) are necessary conditions for the destruction of tumor cells by the immune system [1]. Cytotoxic effects of antigen-specific CTLs against cells of different tumor types have been demonstrated in a number of studies. Back in 2000, it was shown that CTLs specific for HLA-A2-restrictive peptide PR1 could destroy leukemia cells, thus contributing to the elimination of chronic myeloid leukemia [2]. Bernhard et al. demonstrated that CTLs specific for E75 peptide of the HER2/neu tumor antigen could eliminate breast cancer cells in patients after adoptive transfer of HER2-specific CTL populations [3].

Dendritic cells (DCs) are widely used in cancer immunotherapy to stimulate specific antitumor immune responses, because they can effectively recognize and present tumor antigens to T cells both in vitro and in vivo [4–6]. DC vaccines are currently widespread [7]. However, much controversy still surrounds the issue of whether endogenously activated T-cell responses can mediate tumor regression, because tumor progression is often observed even in the presence of high levels of blood-circulating or tumor-infiltrating T cells [8, 9]. Meanwhile, successful ex vivo activation of antitumor cytotoxic T cells (in the absence of immunosuppressive tumor effects) followed by adoptive transfer of activated T cells that eventually retain their ability to eliminate tumor cells in the patient’s body has been reported [3]. This advantage of adoptive transfer of autologous T cells activated ex vivo provided grounds to suggest the promising potential of generating an optimal protocol to obtain antigen-specific CTL populations using DCs loaded with a tumor antigen under in vitro conditions, which can be further used to develop effective antitumor cell-based vaccines.

It is currently possible to isolate CTLs specific for certain antigenic determinants from heterogeneous peripheral blood mononuclear cell (PBMC) populations, thus making it possible to target specific cell-mediated immune responses against tumor cells carrying these antigens [7, 10, 11]. A number of procedures employing MHC molecules have been developed to isolate populations of antigen-specific T cells. The principle of these procedures involving MHC molecules is based on use of a T-cell receptor (TCR) ligand, an MHC/antigen peptide complex, as a dye probe via conjugation of the complex to fluorochrome molecules. Recombinant MHC molecules are conjugated to antigen epitopes typical of various types of disease, such as epitopes of tumor antigens. MHC molecules interact with TCR expressed on the T-cell surface. As TCR–MHC interactions are characterized by very weak affinity for one another, monomeric MHC/epitope complexes cannot ensure stable binding. This problem has been solved using multimeric MHC/epitope complexes, which increase the affinity of the binding reaction, and thus facilitate the formation of a stable complex [12]. Thus, MHC multimers can be used to identify and isolate antigen-specific CTLs.

In 2007 the novel Streptamer technology has been developed for the detection and purification of antigen-specific T cells [13]. A distinctive feature of this technology from other MHC multimer-based technologies is the reversibility of staining which allows complete dissociation of all staining reagents from the cell surface after the isolation procedure. Thus, several side effects caused by long-term presence of staining molecules on the surface of labeled cells might be avoided, including T-cell anergy, immune responses directly against the reagents, and loss of the capacity of the transferred T cells to migrate in vivo. Thereby the Streptamer technology significantly improves the quality of the antigen-specific T-cell populations obtained and makes it possible to use the isolated cells in clinical practice for adoptive T-cell transfer [14].

The membrane protein HER2 (human epidermal growth factor receptor 2: HER2/neu) is a tumor-associated antigen whose overexpression is observed in various types of carcinomas, including breast, colon, stomach, pancreatic, and thyroid carcinomas, as well as ovarian cancer [15]. It is known that 20–25% of breast tumors are characterized by HER2 overexpression. Receptor overexpression or HER2 oncogene amplification are associated with the most aggressive phenotype of this type of tumor, shorter relapse-free period after primary therapy, and worse survival rate [16]. HER2 was shown to be a pathological biomarker that can differentiate between tumor and normal mammary gland cells, because HER2 expression on the surface of HER2-overexpressing breast carcinomas can be up to 50 fold higher than its expression on normal cells [17]. The fundamental role of HER2 in triggering the signaling cascade that results in tumor growth determines the trajectory of development of breast cancer therapies toward targeting this antigen.

Hence, the development of optimized protocol for generating populations of antitumor antigen-specific CTLs using DCs and isolating antigen-specific cells is promising for the most efficient production of T-cell preparations toward adoptive transfer aimed at eliminating the minimal residual disease after removal of the major tumor burden, in the form of tumor cells disseminated in the patient’s body.

This study aimed to develop a protocol for obtaining and enriching populations of antitumor cytotoxic T cells specific for tumor-associated protein HER2 and to assess their cytotoxic activity against HER2-expressing MCF-7 tumor cells. The main advantage of the proposed approach is the combination of protocol of generation of an specific cytotoxic antitumor T cell-mediated immune response by dendritic cells transfected with DNA constructs encoding the immunogenic HER2/neu epitopes and the technology of isolation of activated antigen-specific CTLs which allows rapid and complete removal of the staining reagents from the T cells and thus assures the isolation of fully functional T cells with non-affected viability. Combining methods of efficient generation of antigen-specific antitumor T cell populations and subsequent isolation of activated cytotoxic T cells appropriate for clinical use, this approach can be used to generate functionally active antigen-specific T lymphocytes from peripheral blood mononuclear cells of patients with HER2-positive malignancies for adoptive T-cell transfer to eliminate HER2-positive tumor cells, prevent metastasis and relapse.

## Methods

### Blood samples

Peripheral blood samples from healthy donors carrying the HLA-A02 allele, as shown by genotyping (n = 16), were used in this study. Whole blood samples were obtained from Blood Procurement Station No. 1 of the State-Government-financed Institution of Public Health for the Novosibirsk Region (Novosibirsk Blood Center). Voluntary informed consent was obtained from all the donors. Study design was approved by the local ethics committee of the Research Institute of Clinical Immunology, Siberian Branch of the Russian Academy of Sciences (protocol no. 75 dated April 9, 2013).

### DNA constructs and HER2 protein epitopes

To generate strong immune responses, we selected HER2 protein epitopes E75 (HER2 p369–377; KIFGSLAFL) and E88 (HER2 p689–697; RLLQETELV). These epitopes reported to be most effectively recognized by cytotoxic T cells of HLA-A02-positive donors because of their high affinity for binding to the HLA-A0201 molecule [18–20].

Antigenic activation of mature DCs (mDCs) with HER2 tumor-associated antigens was performed by magnet-assisted transfection with a pMax DNA construct encoding the epitopes E75 and E88 from HER2/neu protein. Proteasomal cleavage and TAP predictions confirmed that flanking sequences are not required for correct processing of selected epitopes. Nevertheless, DNA construct encoding multiple copies of each epitope in different molecular contexts (with or without spacers respectively) was designed for further ensuring a correct antigen presenting and effective stimulation of antigen-specific immune responses by DCs. An ubiquitin was bound to N terminus of prepared DNA construct. The artificial gene encoding poly-CTL epitope immunogen was designed and prepared with an optimization of codon composition for efficient expression in mammalian cells. Next, the artificial gene was cloned into the pmax-Ub vector and resulting DNA construct with N-terminal ubiquitin was prepared. DNA construct was amplified in a preparation form and purified from endotoxins. The nucleotide sequences were verified by sequencing. An additional file shows the resulting amino acid sequences (see Additional file 1).

The construct based on the DNA-vector pDNAVACCultra5 without inserts of immunogenic peptides was used as the control for magnet-assisted transfection of DCs [DNA (p5) construct].

### Donor genotyping

DNA for genotyping was isolated from whole blood samples of the donors using the standard phenol–chloroform extraction procedure [21]. HLA-A locus genotyping was carried out by PCR amplification of gene regions using a commercial ALLSET™ GOLD HLA A LOW RES SSP Kit (Invitrogen, USA) according to the manufacturer’s protocol. We also used Hot Start Taq DNA polymerase (SibEnzyme, Russia). The amplified DNA fragments were identified by 2% agarose gel electrophoresis. DNA molecular weight markers M16, pUC19/Msp I (SibEnzyme, Russia), and Tris-acetate buffer were used for gel electrophoresis.

### Generation of mDCs

RPMI-1640 medium (Biolot, Russia) supplemented with 10% fetal calf serum (Hyclone, USA), 2 mM L-glutamine (Biolot, Russia), 5 × 10^−5^ mM mercaptoethanol (Sigma, USA), 25 mM HEPES (Sigma, USA), 80 μg/ml gentamicin (KRKA, Slovenia), and 100 μg/ml ampicillin (Sintez, Russia) was used for culture of mononuclear cells (MNCs).

Venous blood samples from healthy donors carrying the HLA-A02 allele were collected into lithium heparin vacuum tubes (Vacuette LH Lithium Heparin; Greiner Bio-One GmbH, Austria). PBMCs were isolated using a conventional density gradient of Ficoll–Urografin [22]. Briefly, 12-ml samples of blood diluted in RPMI-1640 medium to a final volume of 35 ml were layered over 15 ml of Ficoll–Urografin solution (ρ = 1.077 g/L) (Ficoll: Pharmacia Fine Chemical, Switzerland; Urografin: Schering AG, Germany), centrifuged at 1500 rpm for 40 min, and washed twice with RPMI-1640 medium. Cells with increased adhesion were isolated by incubation for 2 h on plastic Petri dishes (Nunc, Denmark) in 10 ml of culture medium under a humid atmosphere at 37 °C and 5% CO_2_. After the incubation, the entire medium containing the fraction of non-adherent PBMCs was transferred into clean centrifuge tubes. Next, 10 ml of RPMI-1640 medium was added to the Petri dishes and the fraction of adherent PBMCs was removed from the bottom of the Petri dishes with a scraper (Sigma-Aldrich, USA). The suspensions containing the adherent and non-adherent fractions were centrifuged at 1500 rpm for 10 min. After the centrifugation, the cell pellets were resuspended in 1 ml of culture medium, and the numbers of cells in both fractions were counted in a Goryaev chamber using acetic acid. The non-adherent cell fraction was then cultured in Petri dishes at a concentration of 2 × 10^6^ cells/ml in 10 ml of culture medium for 6 days, until the coculture procedure was started.

To stimulate monocyte differentiation of the adherent fraction of PBMCs into immature DCs (iDCs), the adherent cell fraction at a concentration of 1 × 10^6^ cells/ml was cultured in culture medium in 48-well plates (Cellstar, USA) under a humid atmosphere at 37 °C and 5% CO_2_ in the presence of GM-CSF (50 ng/ml) and IL-4 (100 ng/ml) (Peprotech, USA). After 96 h of culture, the culture medium was replaced and TNFα (25 ng/ml) (State Research Center of Virology and Biotechnology “Vector”, Russia) was added to the iDC culture to stimulate the maturation and generation of mDCs.

### Loading of mDCs with tumor antigen

Antigenic activation of mDCs with tumor-associated antigen HER2/neu was performed by magnet-assisted transfection using magnetic nanoparticles (MATra-A Reagent; PromoKine, Germany), a pMax DNA construct encoding two HER2/neu protein epitopes (E75 and E88) and a DNA (p5) construct (without inserts of immunogenic peptides) as a control. Magnet-assisted transfection was carried out in accordance with the protocol provided by the manufacturer (PromoKine).

To evaluate the effectiveness of transfection into DCs, magnet-assisted transfection of DCs was performed with the pmaxGFP plasmid encoding green fluorescent protein (GFP). The transfection effectiveness was evaluated based on production of the protein encoded by the plasmid. Specifically, the number of GFP-positive cells was determined in a BD FACS Verse flow cytometry system (Becton Dickinson, USA) at 24 h after the magnet-assisted transfection or nucleofection of DCs. Added propidium iodide (PI) (Sigma, USA) allowed determination of the viability of the cell culture post-transfection.

### Phenotyping of DCs and assessment of their functional activity

The phenotype of DCs was assessed by flow cytometry in a BD FACS Aria (Becton Dickinson, USA) using corresponding monoclonal antibodies labeled with fluorochromes (CD3-Pacific Blue, CD14-FITC, HLA-DR-PerCP-Cy5.5, CD11c-PE, CD86-PE-Cy7, and CD83-APC; Becton Dickinson, USA) according to the manufacturer’s recommendations. Examples of the used gates are shown in additional files (see Additional file 2).

The criterion for functional activity of DCs was their susceptibility to receptor-mediated endocytosis by FITC-dextran capture (Sigma, USA). Briefly, the cells were incubated with FITC-dextran (1 μg/ml) in the complete medium at 4 and 37 °C. Dextran became bound to the surface receptors at 4 °C, and the bound dextran penetrated into the cells at 37 °C (endocytosis).

### Generation of activated HER2-specific T cells

To generate HER2-specific T cells, the monocyte-depleted PBMC culture (non-adherent MNCs, 1 × 10^6^ cells/ml) was cocultured with DCs (1 × 10^6^ cells/ml). The coculture was performed at an MNC:DC ratio of 10:1 in the culture medium (1 ml/well) in 48-well plates (Cellstar, USA) for 4 days.

### Streptamer staining and identification of HER2-specific T cells

To identify the population of HER2-specific T cells in the coculture of MNCs and DCs, the cells were stained using MHC I-Strep HLA-A*0201 (plus peptide KIFGSLAFL of the HER2/neu antigen), Strep-tactin PE, and IS buffer (IBA GmbH, Germany). The stained samples were then analyzed by flow cytometry on the BD FACS Verse system. For staining complex formation, 0.8 μl of MHC and 1 μl of Strep-tactin PE were incubated in the IS buffer solution, at a final volume of 25 μl, for 45 min at 4 °C. After incubation of the cells with the complex, the reaction was stopped by adding 200 μl of IS buffer to the reaction medium. The cells were then washed twice in 200 μl of IS buffer and analyzed using the flow cytometry system. Cells incubated in the presence of 1 μl of Strep-tactin PE only were used as a control for nonspecific binding. All stages of the Streptamer staining were conducted at 4 °C.

### Isolation of HER2-specific T cells

HER2-specific T cells were isolated by magnetic-activated cell sorting on MS Columns (Miltenyi Biotec, Germany). HER2-specific T cells were tagged using Strep-Tactin Nano Bead magnetic particles, as well as reagents MHC I-Strep HLA-A*0201 (plus KIFGSLAFL peptide of the HER2/neu antigen), MHC I-Strep HLA-A*0201 (plus RLLQETELV peptide of the HER-2/neu antigen), IS buffer solution, and D-Biotin (IBA GmbH, Germany). Magnetic-activated cell sorting was carried out in accordance with the manufacturer’s protocol with some modifications made by the researchers. To remove magnetic particles from the isolated cells, the eluted fraction of antigen-specific cells were centrifuged at 1400 rpm for 10 min at 4 °C, and the cell pellet was resuspended in 1 ml of 1 mM D-Biotin and incubated for 15 min at 4 °C. Next, the cells were centrifuged at 1400 rpm for 10 min at 4 °C, incubated with 1 mM D-Biotin, and centrifuged again. After resuspension of the cell pellet in 400 μl of culture medium, the cell number and viability were counted in a Goryaev chamber using erythrosine staining. The cells were then transferred into the wells of a 96-well plate (100 μl per well) for further culture.

### Stimulation of proliferation of HER2-specific T cells and evaluation of effectiveness of magnetic-activated cell sorting

To effectively culture the isolated HER2-specific T cells required for further experiments, we carried out a preliminary experiment to select the optimal cytokine concentrations for stimulation of T-cell proliferative activity. The cytokine concentrations were titrated with respect to proliferation of CD8^+^ T cells labeled with CFSE (Molecular Probes, USA) in peripheral blood samples from healthy donors.

To isolate the population of CD8^+^ T cells, PBMCs from healthy donors were stained with anti-CD8-FITC antibody (eBioscience, USA) by incubation for 30 min at room temperature, and washed by centrifugation at 1500 rpm in 1.5 ml of PBS. CD8^+^ cells were isolated by flow cytometry in the BD FACS Aria cell sorter (sorting rate, 5–7 × 10^3^ events per second; sorting effectiveness, 93–95%; purity of sorted cells, 98–99%).

The sorted population of CD8^+^ cells was labeled with CFSE as follows. Cytotoxic T cells (5 × 10^6^ cells) were resuspended in 0.5 ml of 25 mM PBS containing 0.1% BSA, followed by addition of 2 μM CFSE. The cells were incubated with CFSE at 37 °C for 10 min with occasional shaking. Next, a fivefold volume of cold RPMI-1640 medium supplemented with 10% FCS was added and the mixture was centrifuged at 1500 rpm for 10 min. Following the centrifugation, the supernatant was removed and the cells were washed twice with a fivefold excess of cold RPMI-1640 medium supplemented with 10% FCS. After the final wash, the supernatant was removed and the cell pellet was resuspended in the supplemented RPMI-1640 culture medium to a final concentration of 1 × 10^6^ cells/ml.

The CFSE-labeled CD8^+^ T cells were cultured for 5 days at 37 °C under 5% CO_2_. The proliferative activity was analyzed in the BD FACS Verse flow cytometer. The lymphocyte gate was isolated based on the scatter plot with forward and lateral light scattering. In the CFSE fluorescence histogram, the interval gate of cells that had undergone cell division was isolated.

The optimal combination and concentrations of cytokines were selected based on the results for titration of cytokine concentrations as follows. At the culture stage for the required amount of antigen-specific cytotoxic T cells, the target cell fraction after magnetic-activated cell sorting was cultured in a 96-well plate at a concentration of 1 × 10^6^ cells/ml in the culture medium in the presence of rhIL-7 (50 ng/ml), rhIL-15 (50 ng/ml), and rhIL-2 (50 ng/ml) cytokines (Biozol, Germany) for 10–14 days.

After stimulation of proliferation of the isolated cells, the amount of cells carrying the CD8 surface marker was analyzed by labeling the cells with the anti-CD8-FITC antibodies, followed by analysis in the BD FACS Verse flow cytometer.

### Cytotoxicity assay

The human breast adenocarcinoma cell line MCF-7 (cell culture line collection at the Institute of Cytology, Russian Academy of Sciences, St. Petersburg, Russia) was used as the target cells for assessing the direct cytotoxicity of HER2-specific T cells. Frozen MCF-7 cells for the experiment were thawed using a standard procedure, in which the cryoconservation agent was removed by washing. The thawed tumor cells were then cultured in EMEM medium (State Research Center of Virology and Biotechnology “Vector”, Russia) supplemented with 10% FCS, 2 mM L-glutamine, 5 × 10^−5^ mM mercaptoethanol, 10 mM HEPES, 80 μg/ml gentamicin, 100 μg/ml ampicillin, and 10 μg/ml insulin. Prior to use, the tumor cells were subjected to 4–5 passages in culture flasks (Nunc, Denmark) with 5 ml of the culture medium. The cell passages were performed every 3–4 days until a high-density monolayer of tumor cells was obtained. The cells were then detached using trypsin–versene solution, comprising a 1:3 mixture of 0.25% trypsin (Biolot, Russia) and 0.02% versene (Biolot, Russia).

In the cytotoxicity assay, the target cells were tagged with the CFSE label while alive. The tumor cells were detached from the plastic culture flasks using trypsin–versene solution and washed once with EMEM medium, followed by resuspension of the cell pellet in 0.5 ml of 25 mM PBS supplemented with 0.1 BSA. Next, 2 μM CFSE was added to the cell suspension containing ~2–5 × 10^6^ cells, and the mixture was incubated for 10 min in a humid atmosphere at 37 °C in 5% CO_2_.

The tagged cells were then washed twice with 5 ml of ice-cold MCF-7 growth medium and cocultured with HER2-specific T cells at a 1:10 ratio in a 96-well plate in 100 μl of RPMI-1640 medium supplemented with 10% FCS, 2 mM L-glutamine, 5 × 10^−5^ mM mercaptoethanol, 10 mM HEPES, 80 μl/ml gentamicin, and 100 μl/ml ampicillin for 48 h. A coculture of tumor cells and MNCs, and a culture of MCF-7 cells that were not subjected to coculture with MNCs (control over spontaneous death of target cells) were used as controls.

After incubation for 48 h, the cell cultures were tagged with PI. Briefly, 1 μl of PI (1 μg/ml) was added to 100 μl of cell suspension and the resulting mixture was incubated at room temperature for 10 min. The tagged cells in the control and experimental samples were analyzed by flow cytometry in the BD FACS Verse system.

### Statistical analysis

All data were processed using Statistica 7.0 (Dell, Austin, TX, USA). The Wilcoxon test was used to detect statistically significant differences because the statistical samplings exhibited an abnormal distribution. The data are presented as the medians and interquartile ranges. The percentage of proliferating CTLs data are presented as mean and standard error of the mean.

## Results

### Evaluation of the phenotype and functional activity of the resulting DCs

**Fig. 1:**
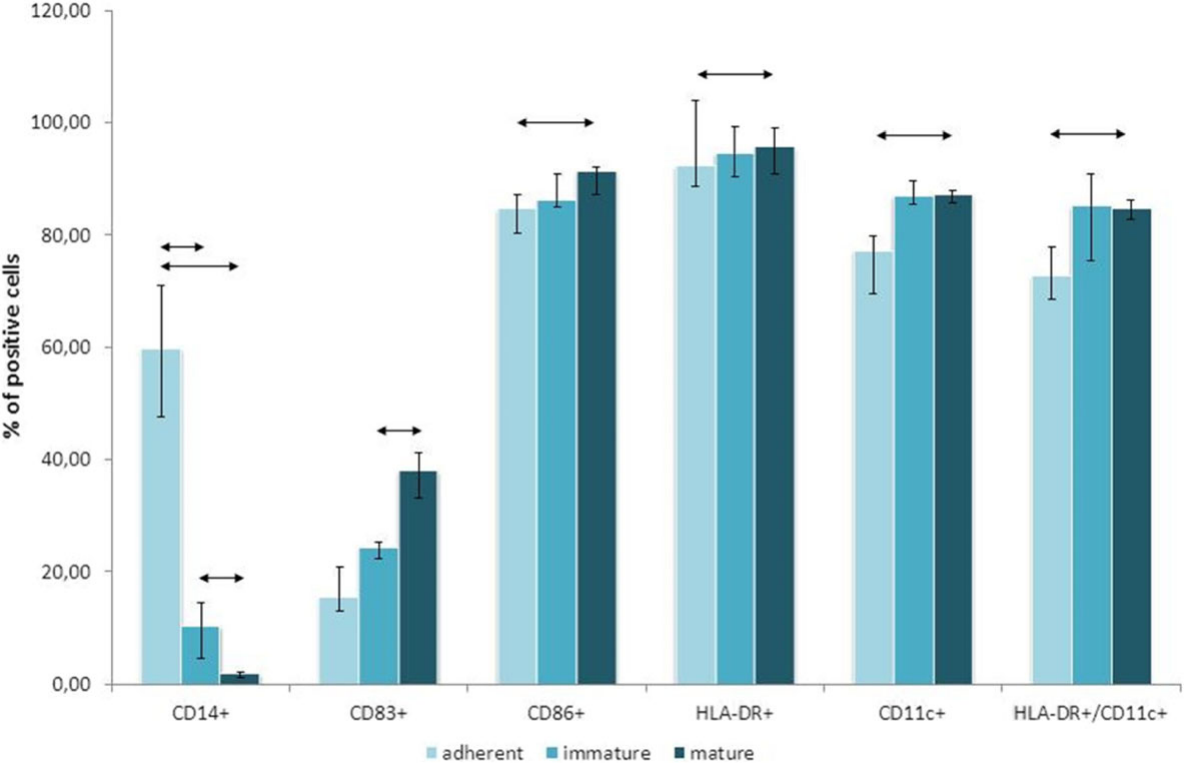
Relative numbers of cells expressing DC markers. DCs were analyzed by flow cytometry in the region of large granular leukocytes. The data are presented as median and interquartile range. *Arrows* show significant differences (*p* < 0.05; *n* = 12)

Phenotyping of adherent MNCs, iDCs, and mDCs using specific antibodies against surface markers described in the literature as DC markers [23–25] was performed to evaluate the effectiveness of the protocol for producing DCs from the adherent fraction of MNCs. DCs were analyzed by flow cytometry in the region of large granular leukocytes. Significant differences in expression of the following markers were demonstrated for the populations of iDCs and mDCs: downregulation of CD14 expression (10.25 and 1.75%, respectively) and upregulation of CD83 expression (24.25 and 37.95%, respectively). In addition, the relative numbers of cells expressing CD86, HLA-DR, CD11c, and HLA-DR/CD11c markers in cultures of mDCs were shown to be reliably increased compared with cultures of the adherent fraction of MNCs (Fig. 1).

The significant increase in the relative numbers of cells expressing the high-specificity mDC marker CD83 and costimulatory molecule CD86 for cells at the mDC stage compared with those at the iDC stage confirms the effectiveness of the protocol for the generation of mDCs. The reliable increase in the percentages of cells expressing DC markers (HLA-DR, CD11c, HLA-DR/CD11c) in the fraction with mDCs compared with the fraction of adherent cells also attests to the effective differentiation of monocytes to DCs. The decrease in the percentages of cells expressing population markers of other immune cells, such as CD14 (marker of monocytes, macrophages, and neutrophils) and CD3 (T-cell marker) as the degree of maturation increases in the series of fractions under investigation demonstrates that DCs, but not other cell populations carrying cell markers common to those of DCs, predominate in the resulting fractions.

Activity with respect to receptor-mediated endocytosis (via FITC-dextran capture) was evaluated at 4 and 37 °C to examine the antigen-capturing ability of the generated DCs. The degree of FITC-dextran capture by DCs was determined using the formula: index of endocytotic activity = (MFI 37 °C/MFI 4 °C) × 100%, where MFI 37 °C is the fluorescence intensity of labeled cells at 37 °C (specific endocytotic activity) and MFI 4 °C is the fluorescence intensity of labeled cells at 4 °C (nonspecific activity).

Mature DCs were shown to exhibit lower activity for antigen capture compared with iDCs, which were capable of more efficient antigen capture via the mechanism of receptor-mediated endocytosis. These findings confirm that myeloid DCs from peripheral blood monocytes undergo directed maturation and differentiation and are consistent with data in the literature [26]. Thus, the protocol used in the present study allows the generation of DCs that have the typical phenotype of the mature stage of antigen-presenting DCs and exhibit proper functional activity.

### Loading of mDCs with tumor-associated antigen

The pMaxGFP plasmid, an analogue of the experimental pMax DNA construct that does not encode HER2 epitopes but does encode GFP protein, was used to assess the transfection efficiency with respect to production of the target protein, by analyzing the relative number of cells producing GFP protein using flow cytometry. After magnet-assisted transfection of DCs by the pMaxGFP plasmid, 31,88 ± 1,93% of the cells were confirmed to express GFP protein [6].

### Assessment of the content of HER2-specific T cells

To reveal the population of HER2-specific T cells, MNC/DC cocultures were stained with antigen-specific reagents. The staining protocol was optimized for samples containing 1 × 10^6^ cells. The optimal ratio between the reagents of the staining complex was selected by the results for titration of the MHC concentration: 1 μl of Strep-Tactin-PE and 0.8 μl MHC in a final volume of 25 μl of IS buffer solution. The reagents of the complex were combined and incubated for 45 min at 4 °C. The cells (1 × 10^6^) were incubated in 25 μl of the final complex at 4 °C for 45 min, washed twice with IS buffer, and analyzed in the BD FACS Verse flow cytometer.

**Fig. 2:**
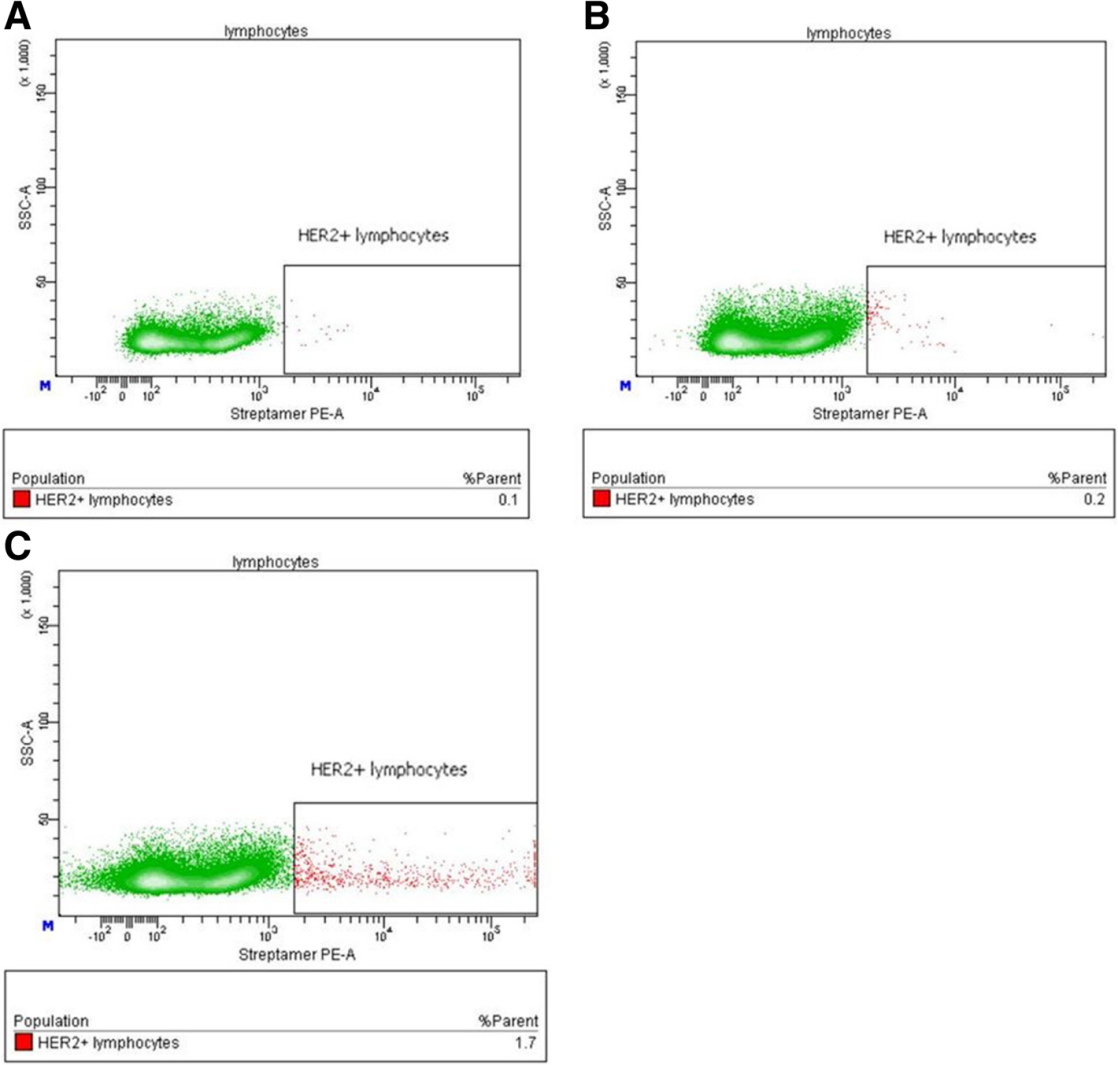
Relative content of HER2-specific lymphocytes in MNC/DC cocultures. **a** Scatter plot showing the distribution of events from lymphocytic region in the nonspecific binding control sample (unstained cells, incubated only in the presence of Strep-tactin PE) is identified. **b** Scatter plot showing the events from the lymphocytic region in the DC-transfection control sample (Streptamer-stained cells of MNC/DC coculture containing DCs transfected with the control DNA (p5) construct without inserts of immunogenic peptides). **c** Scatter plot showing the events from the lymphocytic region in an experimental sample (Streptamer-stained cells of MNC/DC coculture containing DCs transfected with the pMax DNA construct encoding the HER2 protein epitopes)

Cell populations situated in the lymphocytic region were gated according to the parameters of forward (FSC-A) and side (SSC-A) light scattering for analysis. Cells possessing fluorescence in the PE channel corresponding to the population of HER2-specific T cells stained with PE-conjugated Streptamers were then gated from the lymphocytic region, and the percentage of the subpopulation was measured (Fig. 2).

Cytometric analysis of the stained cells revealed that the relative number of HER2-specific lymphocytes in MNC cultures before coculture with antigen-loaded DCs was not higher than the background fluorescence. An analysis of cocultures of MNCs with DCs transfected with the plasmid DNA encoding HER2 protein epitopes and control cocultures of MNCs with DCs transfected with thr DNA (p5) construct showed that the protocol used allowed the production of MNC cultures containing up to 1.7% T cells specific for the E75 epitope (KIFGSLAFL) of the HER2/neu antigen, while control samples contained only 0.1–0.2% (Fig. 2).

### Magnetic-activated sorting of HER2-specific T cells

Magnetic sorting on MS columns was used to isolate the fraction of HER2-specific T cells from cocultures of DCs loaded with tumor antigen and activated MNCs. The manufacturer’s protocol for magnetic-activated cell sorting was mastered for isolation of antigen-specific T cells from cocultures of MNCs and DCs consisting of 0.5–1.0 × 10^7^ cells. The mean number of cells in the sorted fraction of antigen-specific lymphocytes was 0.5–1.0 × 10^5^ cells, because of the low content of HER2-specific T cells in peripheral blood samples from healthy donors. This was also why their number after stimulation with DCs remained low. Induction of proliferation by cytokines IL-2, IL-7, and IL-15 increased the number of antigen-specific cells by 5–10-fold, up to 0.5–1.0 × 10^6^ cells.

**Fig. 3:**
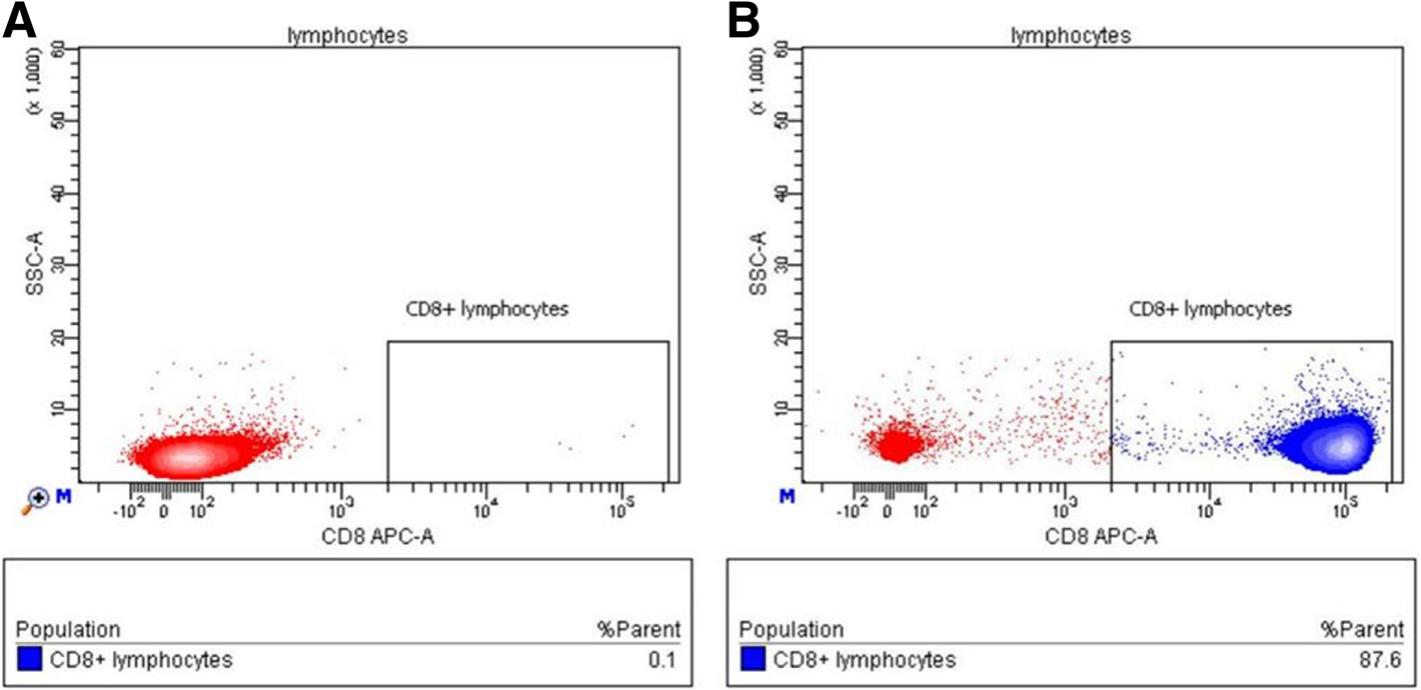
Relative content of CD8^+^ cells in the isolated cultures of HER2-specific T cells (E75-specific cells). The content of CD8+ cytotoxic T cells in isolated fraction was analyzed after culture for 10–14 days in the presence of rhIL-2, rhIL-7, and rhIL-15 stimulants. **a** The scatter plot shows the events from the lymphocytic region in control sample (unlabeled cells). **b** Scatter plot showing the events from the lymphocytic region in an experimental sample (anti-CD8-FITC-labeled cells)

The effectiveness of the magnetic cell sorting procedure was evaluated by analyzing the content of CD8^+^ cytotoxic T cells in the cultures of sorted cells, as direct evaluation was not possible using Streptamer cell staining because of the large number of cells required. An analysis of the cells obtained from eight donors showed that the mean content of CD8^+^ cells was 71.5% in cultures of E88-specific T cells and 90.2% in cultures of E75-specific T cells. Figure 3 shows typical results of the flow cytometry analysis of an experimental sample. Neither monocytic cells (according to the data obtained by forward and side light scattering) nor cells of any morphology other than lymphocytic cells were detected in the experimental sample of antigen-specific cells.

### Enrichment of cultures of sorted HER2-specific T cells

**Fig. 4:**
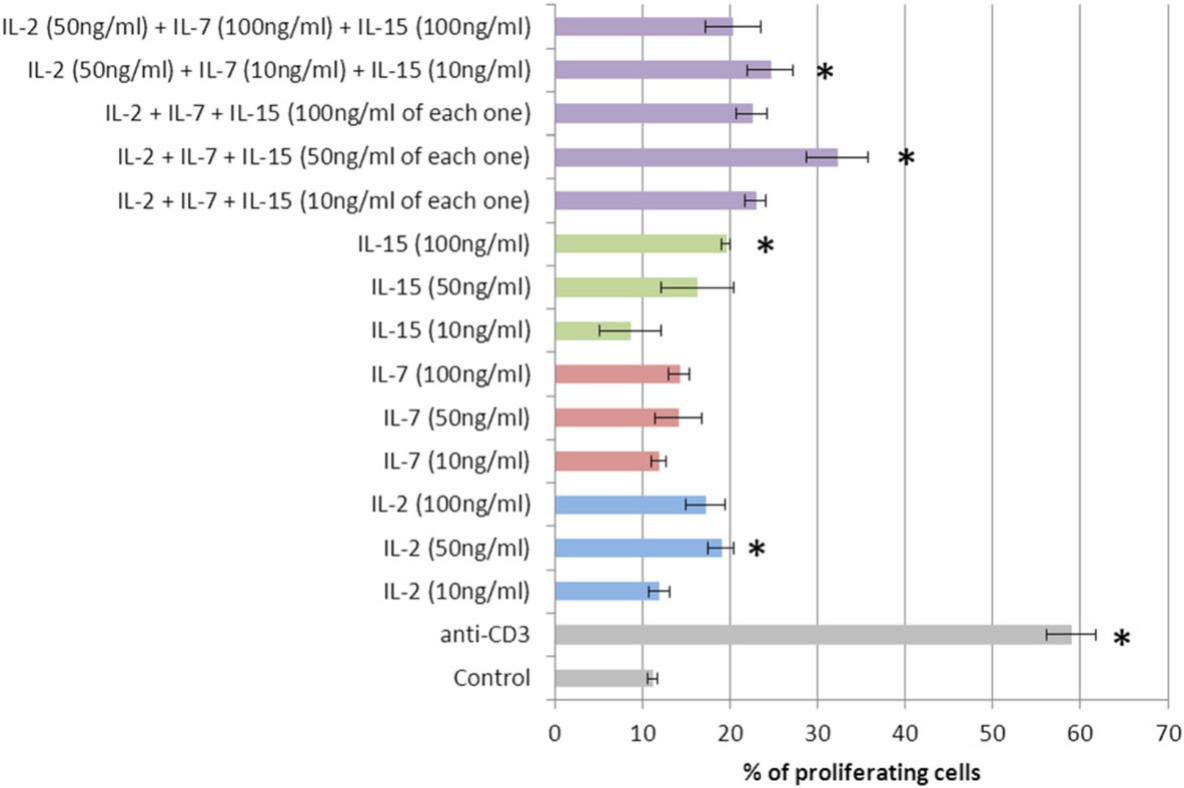
Proliferation of CD8^+^ lymphocytes in the presence of different stimulants (*n* = 6). *p < 0.05, significant difference compared with the control group. Data are presented as mean and standard error of the mean. The column labels show the set and concentrations of stimulants were used. Control – spontaneous CD8+ cell culture. Anti-CD3 – positive control of proliferation (4 μg/ml)

Based on the results for titrating the concentrations of cytokines rhIL-2, rhIL-7, and rhIL-15 and the effectiveness of stimulation of CD8^+^ cell proliferation in cocultures of MNCs in peripheral blood samples from healthy donors, the concentration of 50 ng/ml was selected for each stimulant (Fig. 4). An analysis was carried out using CFSE-labeled cell cultures, in which the numbers of cells that had undergone several divisions were assessed.

### Cytotoxicity assay

Experimental assessment of the cytotoxic effectiveness of the resulting cultures against cells carrying the target antigens on their surface was performed after culture for 10–14 days in the presence of rhIL-2, rhIL-7, and rhIL-15 stimulants.

Specifically, after the antigen-specific cells and target cells were cocultured, the cytotoxic activity of T cells was assessed by flow cytometry according to the percentage of PI-positive tumor cells (MCF-7 cells with damaged membranes) in the samples. Based on the results for titration of cell concentrations, the optimal number of cells per probe was selected: 1 × 10^5^ effector cells and 1 × 10^4^ target cells at a final cell concentration of 1 × 10^6^ cells/ml.

**Fig. 5:**
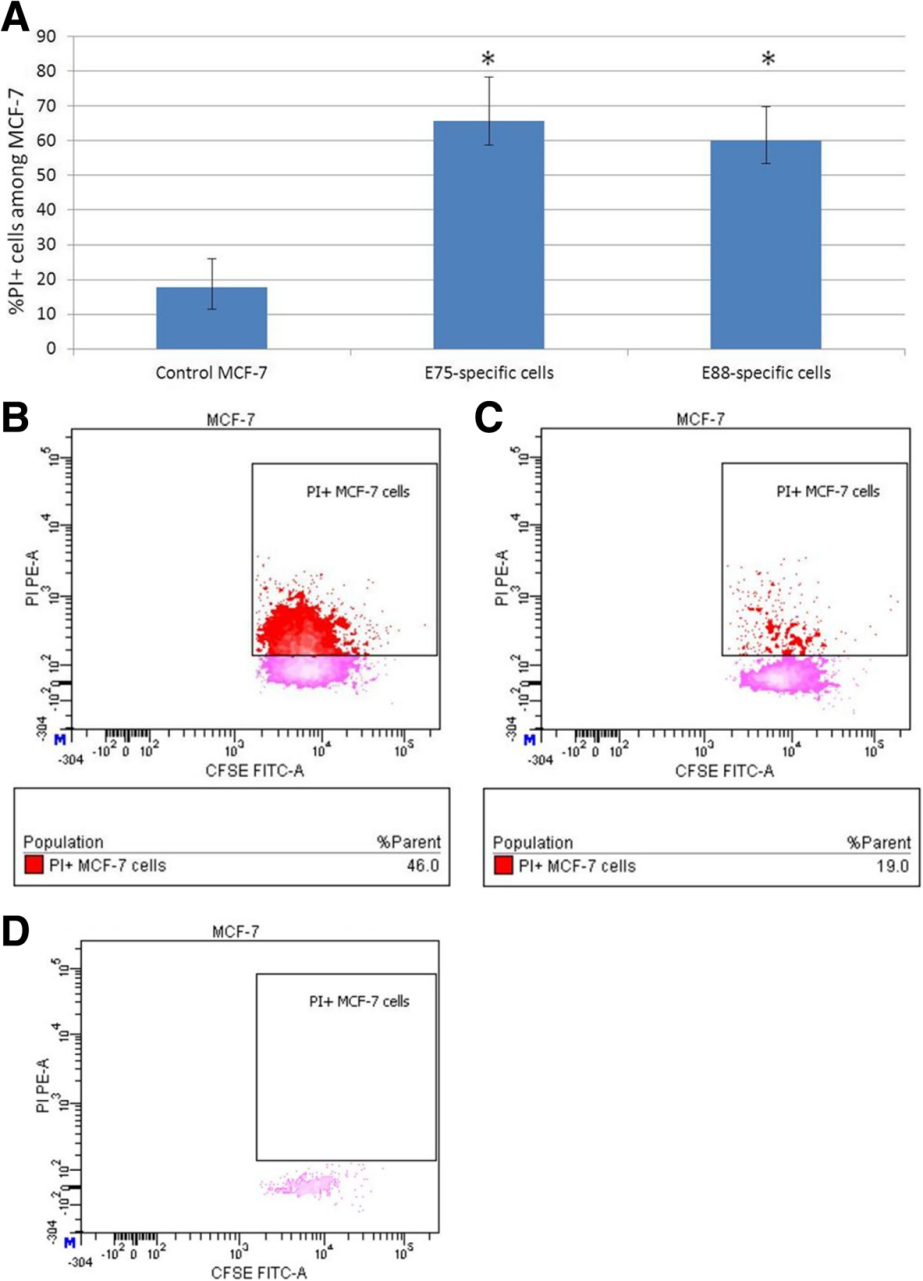
Results of the cytotoxicity assay. **a** Cytotoxic activity of cultures of HER2-specific T cells against the MCF-7 cell line (*n* = 6). Control MCF-7 **–** Control over spontaneous death of the target cells. E75-specific cells **–** Cell culture after magnetic-activated cell sorting at HER2/neu epitope E75 (KIFGSLAFL). E88-specific cells – Cell culture after magnetic-activated cell sorting at HER2/neu epitope E88 (RLLQETELV). *p < 0.05, significant difference compared with the control. Data are presented as median and interquartile range. **b** Scatter plot of the experimental sample (PI-labeled coculture of MCF-7 cells and HER2-specific cells). **c** Scatter plot of the control over spontaneous death of target cells (PI-labeled MCF-7 cells). **d** Scatter plot showing the distribution of events from MCF-7 region in control sample contained MCF-7 cells labeled with FITC but not labeled with PI

The gating scheme involved gating of cells exhibiting fluorescence in the FITC channel, corresponding to the emission parameters of the CFSE label. Next, events corresponding to MCF-7 cells in terms of their phenotypic parameters (forward and side light scattering) were gated among the events in this region. An additional file shows the gating scheme in details (see Additional file 3). An analysis of cell fluorescence from MCF-7 region in the PE channel showed cells with damaged membranes stained with PI (Fig. 5, b – d).

The mean relative number of damaged tumor cells in the test samples consisting of HER2-specific T cells and MCF-7 cells was 60.2% for E88-specific T cells and 65.7% for E75-specific T cells, while the control value characterizing the level of spontaneous death of the target cells was 17.9% (Fig. 5, a).

## Discussion

Cell transfection with plasmid DNA as a means to effectively deliver antigens into DCs is a modern approach to the design of immune therapeutic vaccines in cancer treatment [6, 27]. Currently research groups worldwide are using many different methods for loading of DCs with tumor antigens [6, 28, 29]. In a number of studies, DCs have been loaded by passive capture of soluble immunogenic proteins, or synthetic or eluted peptides [3], coculture of DCs with tumor lysates [28] and delivery of DNA and RNA constructs encoding tumor antigens into DCs [30]. In addition, antigen loading of DCs can be performed using recombinant viral vectors [31], exosomes [32], and apoptotic bodies [33].

We decided to use magnet-assisted transfection of DNA cells with a construct encoding HER2/neu protein epitopes. The use of DNA constructs allows targeted modulation of the immune response against tumor cells expressing a certain antigen, and several genes encoding different antigenic epitopes can be inserted into the DNA plasmid. Different sequences encoding the most immunogenic epitopes can be inserted into the DNA construct, while immunosuppressive fragments of the tumor antigen can be excluded. Moreover, antigenic activation of mDCs generated with a DNA construct encoding immunogenic peptide fragments ensures strong and long-lasting interactions between MHC molecules and the presented antigenic epitopes, thus making it possible to improve antigen presentation to T cells by DCs. In turn, this increases the effectiveness of in vitro generation of antigen-specific T cells.

Sorting of cells carrying TCRs specific for the selected antigenic peptides (epitopes E75 and E88 of the HER2/neu protein) was required to obtain a pure fraction of antigen-specific cells. As the protocol for generating antigen-specific CTLs under development was intended for further use toward designing cell-based drugs for adoptive transfer to cancer patients, we needed to use a technology for sorting antigen-specific cells that would enable the production of target cells that did not carry any staining molecules or magnetic beads on their surface, as their presence is not permitted in a patient’s body. According to data in the literature, the Streptamer technology [13] is currently the optimal method, because it allows complete dissociation of staining reagents from the cell surface. A number of studies have confirmed that TCRs return into their inactivated state, while the viability and functions of the isolated cells are retained after the staining and isolation procedure [14, 34–36].

Sorting of antigen-specific cells using Streptamer reagents can be performed by flow cytometry or magnetic cell sorting. A high sorting speed is one of the benefits of using flow cytometry compared with magnetic cell sorting. However, as HER2-specific CTLs comprise a rare and rather small cell population in PBMC cultures from healthy donors, the flow-through sorting procedure would be associated with higher damage to the target cells caused by high pressure resulting from the high sorting speed. Meanwhile, the magnetic sorting procedure has no noticeable negative effect on the viability of the target cells, because the cellular suspension being sorted passes through a column by gravity acting on the fluid drops, without any additional pressure applied in the system. We took these features into account and selected magnetic sorting as the method for isolating our target cells.

The obtained high percentage of isolated cells carrying the CD8 marker is indirectly indicative of the effectiveness of the magnetic-activated cell sorting procedure. The findings that neither monocytic cells nor cells of any morphology other than lymphocytic cells were detected in the experimental sample of antigen-specific cells also attest to the purity of the resulting population. Furthermore, the magnetic sorting procedure was based on the interactions between magnetic beads conjugated to class I MHC molecules carrying the target epitopes and antigen-specific TCRs. Hence, all of these findings provide grounds for stating that the isolated population was indeed composed of CD8^+^ HER-specific T cells.

The experimental findings demonstrated that resulting antigen-specific CTL populations displayed strong cytotoxic effects against the MCF-7 cell line We argue that the death of MCF-7 cells was mediated by the cytotoxic action of T cells, because the samples under investigation contained only tumor cells and the fraction of antigen-specific T cells, thereby ruling out possible roles of other factors and taking into account the control samples for spontaneous death of the target cells. In this study, we have demonstrated that the generated T-cell fractions possessed cytotoxic properties against MCF-7 cells. Cells of this line are morphologically large and significantly different from tumor cells that develop in vivo. Hence, a hypothesis can be put forward that the cytotoxic effect on autologous tumor cells will be more pronounced.

## Conclusions

A method for generating antitumor cytotoxic T cells specific for the epitopes of the HER2/neu tumor-associated antigen is proposed in this study. The method includes stages of generation and magnet-assisted transfection of mDCs by a DNA construct encoding two immunogenic epitopes of the HER2/neu tumor-associated protein, coculture of antigen-loaded DCs with autologous MNCs, identification and isolation of populations of HER2-specific CTLs from MNC/DC cocultures using reversible Streptamer staining, and enrichment of the isolated cells. The present findings provide grounds for claiming that the designed protocol allows the generation of antigen-specific CTLs that have a pronounced cytotoxic effect with respect to tumor cells expressing the HER2/neu marker.

## Additional files

Additional file 1: The amino acid sequences of pMax DNA construct with N-terminal ubiquitin. N-terminal ubiquitin is underlined; C-terminal G replaced by V in ubiquitin for the protease cleavage site elimination. (DOCX 140 kb)

Additional file 2: Typical scatter plots of gates used in DCs phenotyping analysis. DCs were analyzed by flow cytometry in the region of large granular leukocytes. A – Events corresponding to large granular leukocytes in terms of their phenotypic parameters (forward and side light scattering) are gated. B – Events corresponding CD14-FITC-labeled cells are gated. C – Events corresponding CD83-APC-labeled cells are gated. D – Events corresponding CD86-PE-Cy7-labeled cells are gated. E – Events corresponding double-positive HLA-DR-PerCP-Cy5 and CD11c-PE-labeled cells are gated. (DOCX 785 kb)

Additional file 3: Typical scatter plots of the experimental sample showing the gating scheme. The experimental sample is the propidium iodide (PI)-labeled coculture of MCF-7 cells and HER2-specific cells. A – Scatter plot showing the distribution of all events corresponding to the emission parameters of the CFSE label in the FITC channel. Events corresponding CFSE-labeled cells are gated. B – Scatter plot showing the distribution of events from region of CFSE-labeled cells. Events corresponding to MCF-7 cells in terms of their phenotypic parameters (forward and side light scattering) are gated. C – Scatter plot showing the distribution of all events in the experimental sample with respect to parameters of forward and side light scattering. D – Scatter plot of the experimental sample (PI-labeled co-culture of MCF-7 cells and HER2-specific cells). E – Scatter plot of the control over spontaneous death of target cells (PI-labeled MCF-7 cells). (DOCX 160 kb)

## Abbreviations

CTLs: Cytotoxic T lymphocytes
DCs: Dendritic cells
HER2: Human Epidermal growth factor Receptor 2, HER2/neu
iDCs: Immature dendritic cells
mDCs: Mature dendritic cells
MNCs: Mononuclear cells (meaning monocyte-depleted non-adherent fraction of peripheral blood mononuclear cells)
PBMCs: Peripheral blood mononuclear cells
PI: Propidium iodide
