# Supplementary figures and images for: Generation of populations of antigen-specific cytotoxic T cells using DCs transfected with DNA construct encoding HER2/neu tumor antigen epitopes
Source: BMC Immunol. 2017 Jun 20;18:31. doi: 10.1186/s12865-017-0219-7 (PMC5479015; doi:10.1186/s12865-017-0219-7)

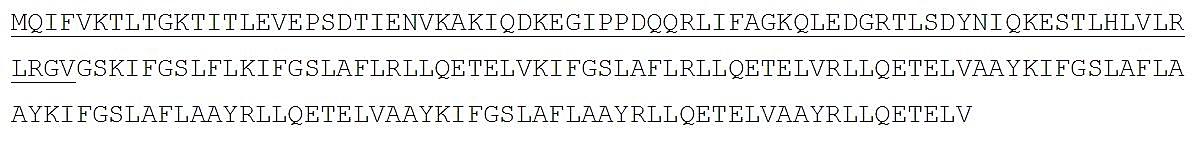

Supplement: Supplementary file 1 — The amino acid sequences of pMax DNA construct with N-terminal ubiquitin. N-terminal ubiquitin is underlined; C-terminal G replaced by V in ubiquitin for the protease cleavage site elimination. (DOCX 140 kb) [file 12865_2017_219_MOESM1_ESM.docx]

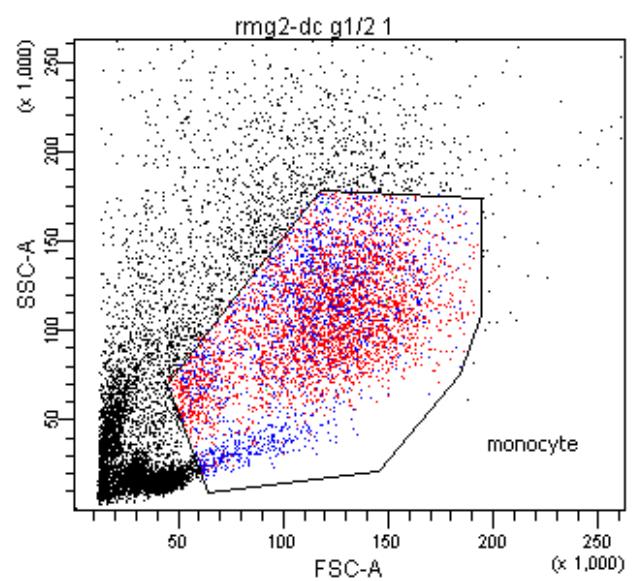

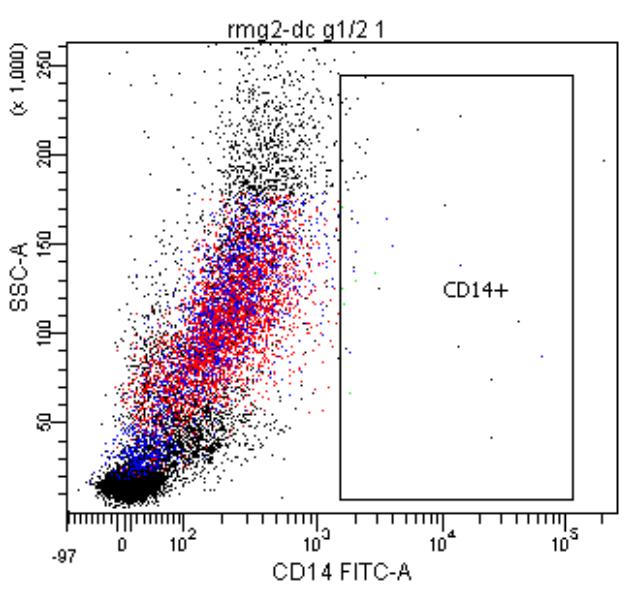

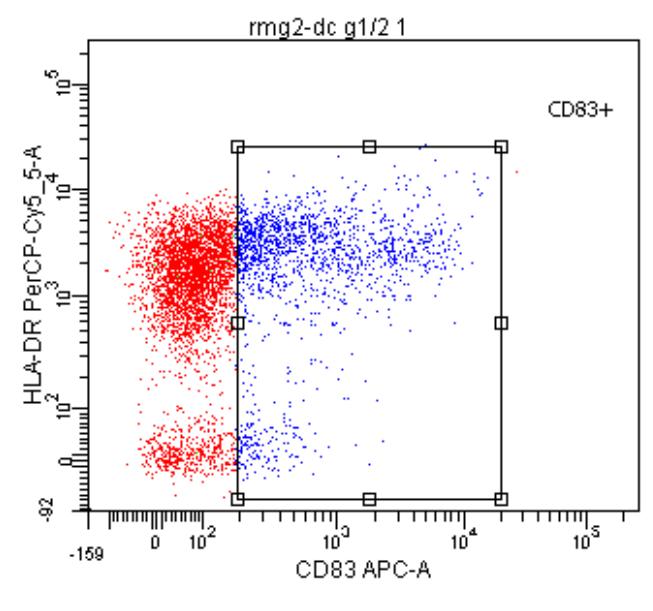

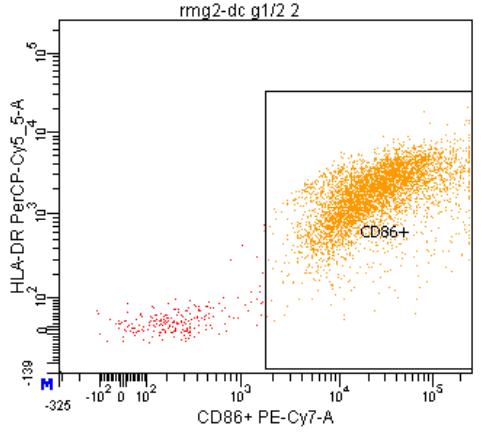

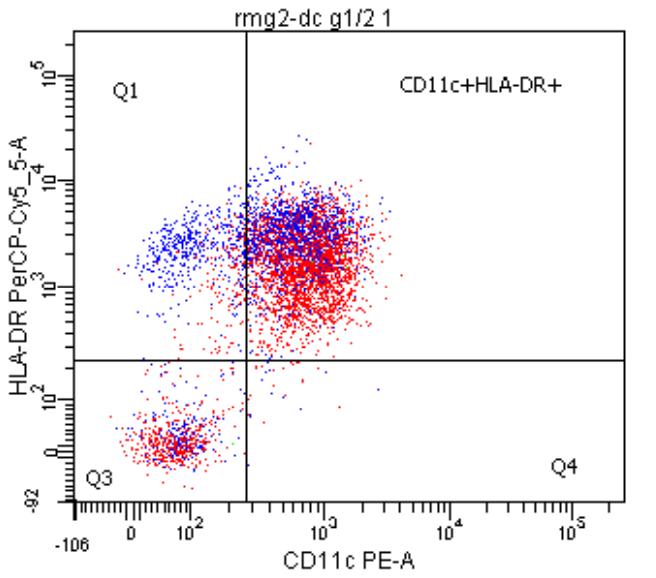

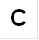

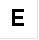

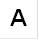

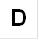

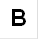

Supplement: Supplementary file 2 — Typical scatter plots of gates used in DCs phenotyping analysis. DCs were analyzed by flow cytometry in the region of large granular leukocytes. A – Events corresponding to large granular leukocytes in terms of their phenotypic parameters (forward and side light scattering) are gated. B – Events corresponding CD14-FITC-labeled cells are gated. C – Events corresponding CD83-APC-labeled cells are gated. D – Events corresponding CD86-PE-Cy7-labeled cells are gated. E – Events corresponding double-positive HLA-DR-PerCP-Cy5 and CD11c-PE-labeled cells are gated. (DOCX 785 kb) [file 12865_2017_219_MOESM2_ESM.docx]

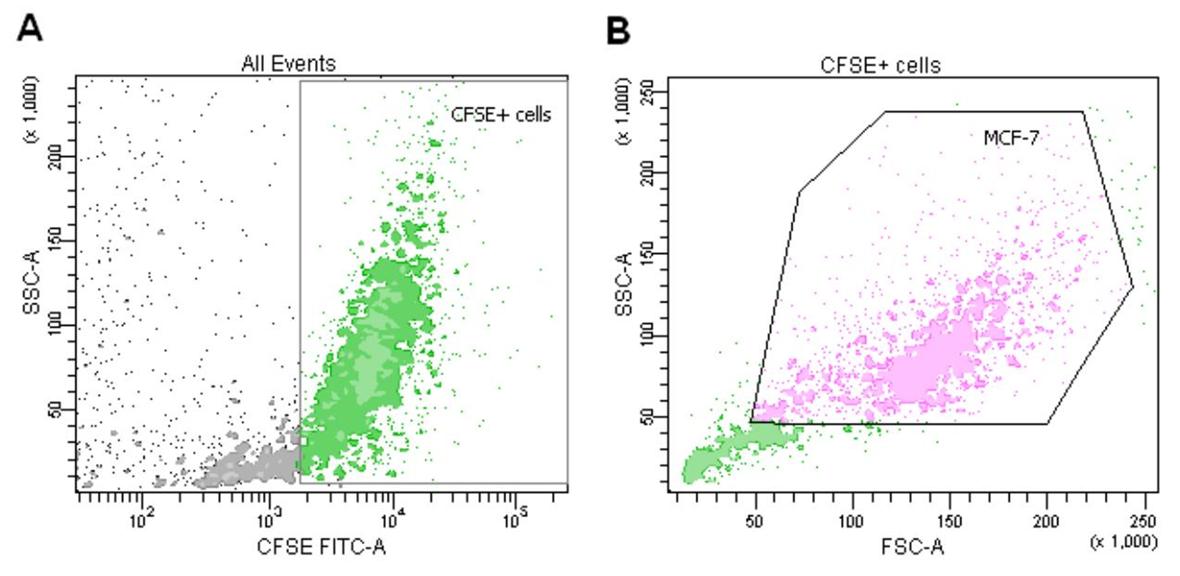


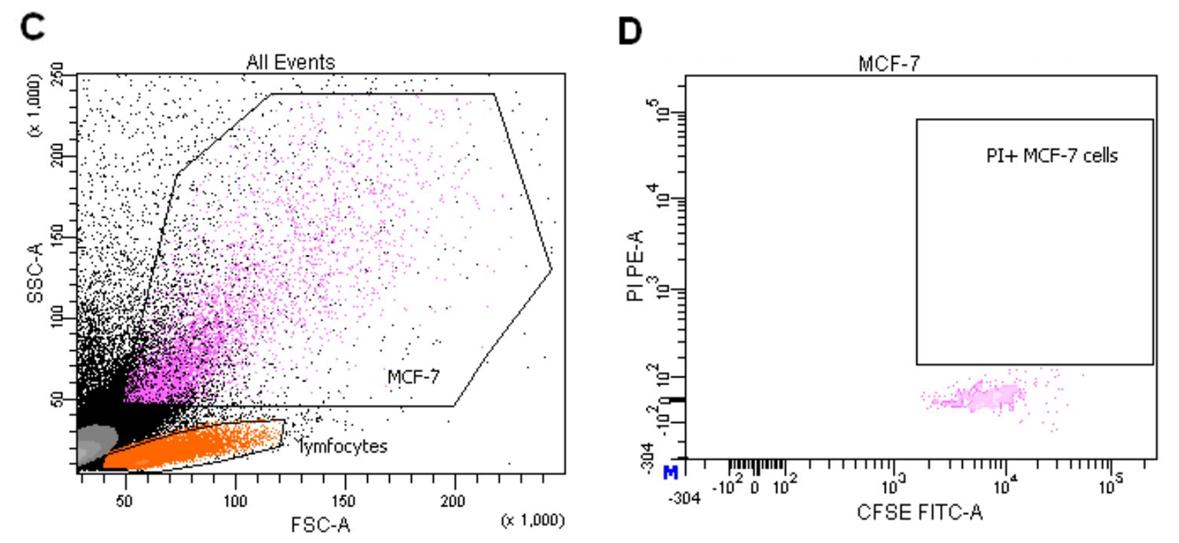

Supplement: Supplementary file 3 — Typical scatter plots of the experimental sample showing the gating scheme. The experimental sample is the propidium iodide (PI)-labeled coculture of MCF-7 cells and HER2-specific cells. A – Scatter plot showing the distribution of all events corresponding to the emission parameters of the CFSE label in the FITC channel. Events corresponding CFSE-labeled cells are gated. B – Scatter plot showing the distribution of events from region of CFSE-labeled cells. Events corresponding to MCF-7 cells in terms of their phenotypic parameters (forward and side light scattering) are gated. C – Scatter plot showing the distribution of all events in the experimental sample with respect to parameters of forward and side light scattering. D – Scatter plot of the experimental sample (PI-labeled co-culture of MCF-7 cells and HER2-specific cells). E – Scatter plot of the control over spontaneous death of target cells (PI-labeled MCF-7 cells). (DOCX 160 kb) [file 12865_2017_219_MOESM3_ESM.docx]
